# Ammonium Production off Central Chile (36°S) by Photodegradation of Phytoplankton-Derived and Marine Dissolved Organic Matter

**DOI:** 10.1371/journal.pone.0100224

**Published:** 2014-06-26

**Authors:** Angel Rain-Franco, Claudia Muñoz, Camila Fernandez

**Affiliations:** 1 Graduate Program in Oceanography, Department of Oceanography, University of Concepción, Concepción, Chile; 2 Department of Oceanography, COPAS SURAUSTRAL program and Interdisciplinary center for Aquaculture Research (INCAR), University of Concepción, Concepción, Chile; 3 Sorbonne Universités, UPMC Univ Paris 06, UMR 7621, Laboratoire d’Océanographie Microbienne, Observatoire Océanologique, Banyuls/mer, France; 4 CNRS, UMR 7621, Laboratoire d’Océanographie Microbienne, Observatoire Océanologique, Banyuls/mer, France; Glasgow University, United Kingdom

## Abstract

We investigated the production of ammonium by the photodegradation of dissolved organic matter (DOM) in the coastal upwelling system off central Chile (36°S). The mean penetration of solar radiation (Z1%) between April 2011 and February 2012 was 9.4 m, 4.4 m and 3.2 m for Photosynthetically Active Radiation (PAR; 400–700 nm), UV-A (320–400 nm) and UV-B (280–320 nm), respectively. Ammonium photoproduction experiments were carried out using exudates of DOM obtained from cultured diatom species (*Chaetoceros muelleri* and *Thalassiosira minuscule*) as well as natural marine DOM. Diatom exudates showed net photoproduction of ammonium under exposure to UVR with a mean rate of 0.56±0.4 µmol L^−1 ^h^−1^ and a maximum rate of 1.49 µmol L^−1 ^h^−1^. Results from natural marine DOM showed net photoproduction of ammonium under exposure to PAR+UVR ranging between 0.06 and 0.2 µmol L^−1 ^h^−1^. We estimated the potential contribution of photochemical ammonium production for phytoplankton ammonium demand. Photoammonification of diatom exudates could support between 117 and 453% of spring-summer NH_4_^+^ assimilation, while rates obtained from natural samples could contribute to 50–178% of spring-summer phytoplankton NH_4_^+^ requirements. These results have implications for local N budgets, as photochemical ammonium production can occur year-round in the first meters of the euphotic zone that are impacted by full sunlight.

## Introduction

Since the discovery of decreasing concentrations of stratospheric ozone over the Antarctic, high levels of incident harmful solar ultraviolet radiation (specifically UV-B) have been a constant feature over the southern hemisphere, mainly during spring. The size of the ozone hole reached a historical maximum in 2006 while unprecedented low levels have also been reported over the arctic [1]. In mid-latitudes, ozone concentrations are currently 6% lower than the long term average for the area [2].

The impact of the different solar spectra in the ocean can lead to deleterious effects on plankton communities [3]–[7]. However, it is also possible to detect “positive” effects of exposure to solar radiation. For instance, the photo-dissociation of dissolved organic matter (DOM) may increase the bioavailability of dissolved organic carbon (DOC) for bacterial growth, potentially stimulating carbon transfer towards higher trophic levels via the microbial loop [8]–[11]. Previous studies on the photochemical production of organic and inorganic compounds of low molecular weight via exposure of DOM to UV radiation (UVR) showed the importance of this mechanism for marine microbial activity [8], [9], [12]–[14]. It is now known that the effect of UVR on DOM can generate, among others, compounds such as carbon monoxide [15], ammonium (NH_4_^+^) [12], amino acids (such as glutamine and alanine), nitrite (NO_2_^−^) and urea [12], [13], [16]–[19]. However, the potential contribution of photoammonification could vary significantly among marine biomes [12], [14], [18], [20]–[22]. UVR could increase by 20% the availability of dissolved inorganic nitrogen (DIN) via ammonium production (photoammonification) in rivers in the southeastern continental shelf of the United States [12]. Additionally, other studies estimated that photoammonification can represent 50% of phytoplankton demand on the Orinoco River plume [22] while meeting 12% of the estimated annual phytoplankton demand (in terms of new N) in the oligotrophic Eastern Mediterranean Sea [14].

The upwelling-system off central Chile (36°S; 73°W) in the Humboldt Current System (HCS) is one of the most productive areas of the world ocean [23]. The observed biological production in this system is supported by the assimilation of new nitrogen (as nitrate injected into the euphotic zone by mixing and vertical advection during seasonal upwelling events) and regenerated nitrogen (derived from in situ remineralization of organic matter that results in NH_4_^+^ release [24], [25]). Additionally, ammonium assimilation by phytoplankton is persistent throughout the year representing almost half of nitrate uptake in active upwelling conditions [26]. Recurrently high concentrations of ammonium off central Chile are also thought to sustain intense chemosynthetic activity via nitrification [27], particularly within the euphotic zone and oxycline [26].

The aim of this study was to evaluate the potential effect of solar radiation, both Photosynthetically Active Radiation (PAR; 400–700 nm) and UV spectra (280–400 nm) in the coastal zone off central Chile (36°S) by quantifying the production of ammonium via photodegradation of marine DOM using exudates two cultured diatom species as well as natural DOM samples.

## Methods

**Figure 1:**
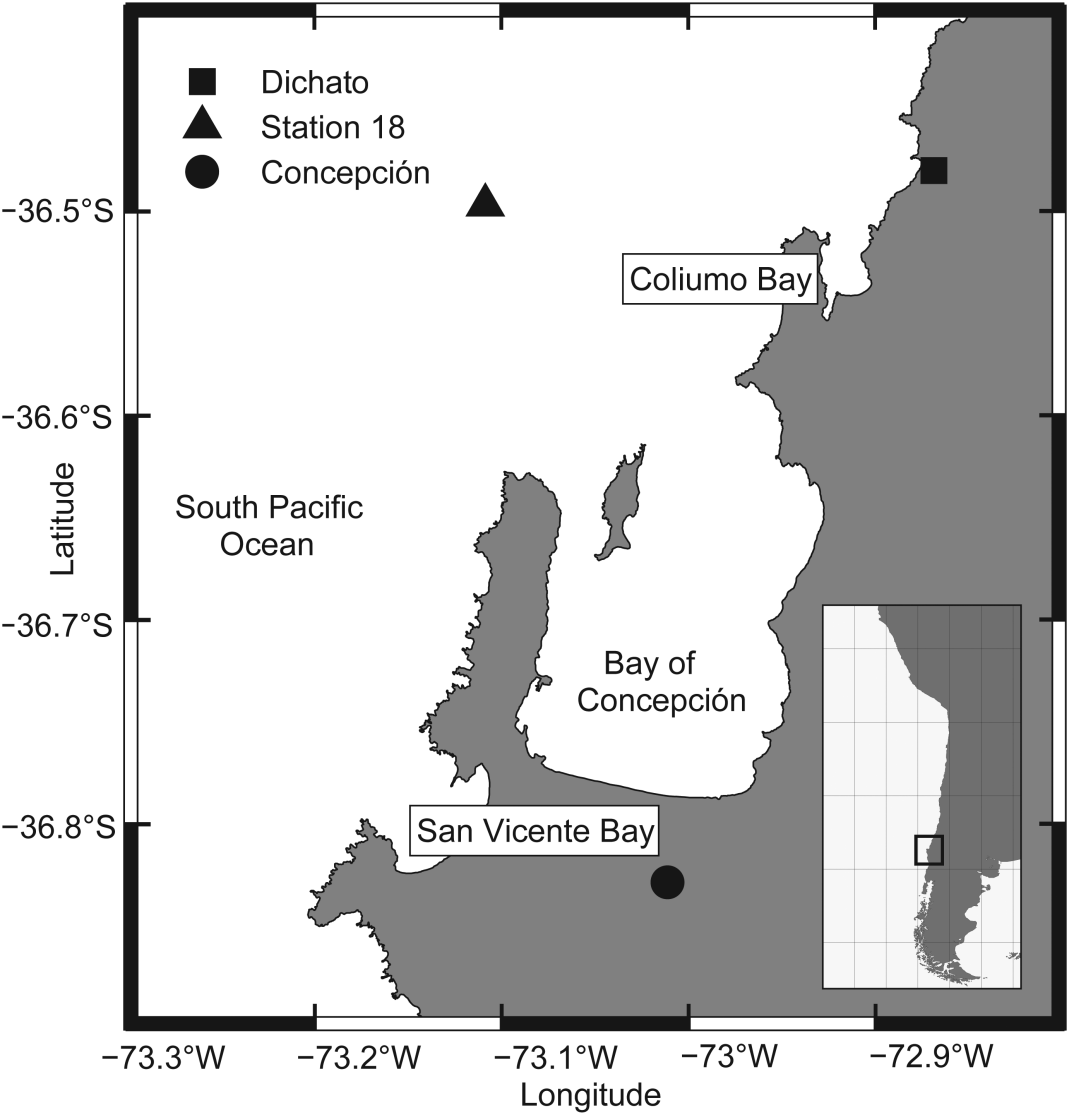
Study area. Location of COPAS Time Series station 18 (*). The sites of atmospheric irradiance measurements (Concepción,•) and light penetration measurements in the water column (Coliumo Bay, Δ) are also indicated.

During this study we focused on two sites located in an active coastal upwelling system off central Chile (36°S). Ammonium photoproduction experiments were performed with sea water collected from station 18 of the COPAS Time Series program. However, schedule restrictions prevented the use of radiometers for incident solar radiation profiling at this station. Station 18 (36° 30.800′S, 73° 7.750′W; Fig. 1) has been studied by the time series program of the COPAS center since 2000 [28]. Samplings for this study were performed in the frame of this time series program with official support of the Chilean Navy (Directemar). Atmospheric measurements of incident solar radiation were carried out in the Concepción area (36° 49.678′S 73° 21.784′W; Fig. 1) while estimations of the depth of solar penetration in the water column were carried out via underwater measurements at Coliumo Bay off the upwelling system (36° 49.669′S 73° 02.162′W; Fig. 1). Although field samplings involved plankton and water chemistry only, this study did not involve endangered or protected species.

### a) Incident Solar Radiation measurements

We carried out atmospheric measurements of incident solar radiation twice per month at noon between April 2011 and February 2012 using a portable radiometer (RM-21 Dr. Gröbel, Germany) equipped with sensors for three spectral ranges: UV-B (defined hereafter as 280–320 nm), UV-A (defined hereafter as 320–400 nm) and PAR (defined hereafter as 400–700 nm). Values of PAR, UV-A and UV-B radiation are expressed in Wm^−2^.

The same instrument was used for estimating the penetration of solar radiation in the water column. Measurements were done under calm weather and low wind conditions every 3 weeks. The depth of penetration of the three spectra was estimated by measuring each spectrum at two depths: 0 m (immediately below the surface) and at 0.3 m depth. The coefficient of vertical light attenuation (Kd in m^−1^) for all spectra was calculated according to Eq. (1):
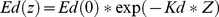
(1)Where Ed _(Z)_ is the irradiance at depth z and Ed _(0)_ is the irradiance just below the surface of the water column [29].

The depth of 1% penetration of incident surface irradiance (Z1%) was calculated for all wavelength spectra (PAR, UVR) by 2.3/Kd. Integrated values of PAR, UV-A and UV-B were calculated by numerically integrating (trapezoidal method) radiation values between the surface and Z1% and will be expressed in Wm^−1^.

### b) Photoammonification from diatom-derived and marine DOM

We performed experiments in order to evaluate the production of ammonium via photo-transformation of DOM using representative diatom cultures with different cell densities (Table 1). Experiments were carried out between September 2011 and April 2014 (Table 1 and 2).

**Table 1:** Summary of ammonium production experiments carried out with exudates of cultured marine diatoms.

Typesample	Cell density(cell mL^−1^)	Date	Dilutionfactor	Exposure(h)	Doses (Kj m^−2^)		
					PAR	UV-A	UV-B
*C. muelleri*	2.49E+06	23/04/2014	1.8	5	243.2	353.0	9.0
*C. muelleri*	2.52E+06	11/10/2011	4	5	261.8	354.9	7.7
*C. muelleri*	3.66E+06	23/04/2014	1.8	5	243.2	353.0	9.0
*C. muelleri*	3.97E+06	21/09/2011	4	2	105.0	142.1	2.8
*C. muelleri*	4.07E+06	24/04/2014	1.8	5	37.2	200.0	540.4
*C. muelleri*	4.44E+06	11/11/2011	2	5	N/A	190.4	582.4
*T. minuscule*	2.82E+06	11/10/2011	1.5	5	261.8	354.9	7.7
*T. minuscule*	3.41E+06	23/01/2012	2	4	N/A	152.6	465.5
*T. minuscule*	3.65E+06	11/11/2011	2	5	N/A	190.4	582.4

A dilution factor was applied to the initial ammonium concentrations since cultures were diluted in milliQ water before incubation in order to ensure volumes necessary to perform the experiment. Doses received by samples for each range of exposure (PAR, UVA, UVB) are described in Kj m^−2^. N/A: Not applied.

**Table 2:** Summary of ammonium production experiments with natural marine samples carried out during this study.

			Dose (KJ m^−2^)		
Date	Depth (m)	Exposure time (h)	PAR	UV-A	UV-B
13/09/2011	5	4	299	406	9
21/11/2011	5	4	N/A	218	665
11/01/2012	5	4	299	406	9

Doses received by samples for each range of exposure (PAR, UVA, UVB) are described in Kj m^−2^. N/A: Not applied.

DOM was obtained from exudates of two cultured species dominant in the study area, *Chaetoceros muelleri* (Lemmermann, 1898) and *Thalassiosira minuscule* (Krasske, 1841). Cultures were maintained in Walne + Si media and filtered through precombusted GF/F filters (0.7 µm, Millipore; 450°C for 6 h). Filtrates containing diatom exudates were diluted previous to incubation using milli-Q water (Table 1) in order to obtain the volume required for performing the experiment (see Table 2). Samples were then distributed in 500 mL quartz bottles (mean transmittance of 70% between 280 and 700 nm) and irradiated with either full solar radiation (PAR+UVR) or UVR only (including UV-A+UV-B; Table 1). Dark control samples were incubated in darkened 500 mL Duran Schott bottles.

All experiments were performed using an irradiation chamber (UV Chamber B-03, Dr Gröbel, Germany) equipped with either PAR+UV (280–700 nm) or UV-A and UV-B lamps (280–400 nm). This instrument is equipped with an internal temperature control system allowing low temperature variations during the experiments. Table 1 summarizes the doses received by all samples. During all incubations, doses of PAR, UVA and UVB were generally within ranges of previously reported winter solar radiation for the study area [30]. We however acknowledge that spring and summer irradiance may be underestimated.

Samples for ammonium determination (20 mL) were taken in triplicate and stored in the dark at room temperature after addition of 5 mL of phthaldialdehyde for fluorometry (OPA). Samples were analyzed by the fluorometric method [31] using a Turner Design fluorometer.

Another set of experiments was designed to evaluate the production of ammonium from marine DOM using natural seawater samples. Seawater (25 L) was taken at 5 m depth at the COPAS time series station 18 (R/V Kay Kay II, Table 2 and Fig. 1). During each sampling, a conductivity temperature depth cast was done (CTD; SeaBird) in order to determine the structure of the water column. The depth of the mixed layer (MLD) was estimated using the thermal criterion (0.2°C) [32].

Experiments were carried out during austral spring and summer (September 2011 - January 2012). Water samples were filtered through pre-combusted GF/F filters (0.7 µm; 450°C, 6 h) using a peristaltic pump. Filtrates were distributed in autoclaved 500 mL glass bottles (Duran Schott for dark control) or 500 mL quartz bottles (UV-A+UV-B treatment). The time of exposure and doses received by all samples are summarized in Table 2.

Samples for ammonium (in triplicate) and bacterioplankton abundance were taken before incubation (T0) and every 2 h. Ammonium determination was carried out as described in the previous section. Determination of nitrite (NO_2_^−^) and nitrate (NO_3_^−^) was made in duplicate in 10 mL samples, which were frozen until laboratory analysis using a standard colorimetric automatic technique (Bran Luebbe autoanalyzer). Bacterioplankton abundance was determined by flow cytometry according to Marie et al (2000) [33]. Samples (1350 µL) were taken in duplicate in sterile cryovials, fixed with glutaraldehyde (at 0.1% final concentration) and stored at −80°C until laboratory analysis at PROFC laboratory at University of Concepcion, Chile.

During all incubations, doses of PAR, UVA and UVB were generally within ranges of previously reported winter solar radiation for the study area [30]. We therefore acknowledge that spring and summer irradiance may be underestimated.

### c) Quantifying net ammonium photoproduction

In order to accurately estimate ammonium photoproduction, we took into consideration the presence of bacterioplankton in samples filtered through 0.7 µm. We therefore assumed that in situ regeneration of ammonium can occur during the incubation, which was estimated for samples incubated in dark conditions. Consequently, we established the following assumptions: 1) The exposure of DOM samples to UVR or PAR+UV radiation always results in ammonium production (other labile N compounds are not taken into account) 2) Photolysis of DOM only occurred under exposure to UVR and was absent in dark controls or PAR exposed samples 3) Complete degradation of DOM leading to limitation does not occur during the experiments 4) Bacterial ammonium “regeneration” is constant during the incubation. Based on these assumptions we propose Eq. (2) for estimating the ammonium production by photolysis of the DOM. Equation (2) evaluates the net change in the ammonium concentration through exposure to PAR+UV/UVR, while taking into account the simultaneous ammonium production that takes place via remineralization of DOM by bacterioplankton activity or ammonium consumption.
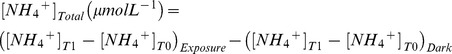
(2)The term [NH_4_^+^]_T0_ represents the ammonium concentration at the beginning of the incubation. The term [NH_4_^+^]_T1_ represents the ammonium concentration at the end of the incubation. The term [NH_4_^+^]_Total_ represents total ammonium production via photolysis. [NH_4_^+^]_Total_ divided by the time of incubation give us the rate of ammonium photoproduction. The sub-indexes “Exposure” and “Dark” identify exposed samples from the dark controls.

Data of ammonium concentration during photodegradation of DOM for each exposure treatment and dark control were analyzed by a paired t-test.

Data of ammonium concentration and bacterioplankton abundance of natural DOM experiments were analyzed by a one-way ANOVA after checking for normality assumption (Kolmogorov-Smirnov test, α = 0.05) and homoscedasticity (Cochran test, α = 0.05). Pairwise multiple comparison were performed using the Tukey test as an *a posteriori* test (α = 0.05).

## Results

### a) Incident solar radiation in the study area

**Figure 2:**
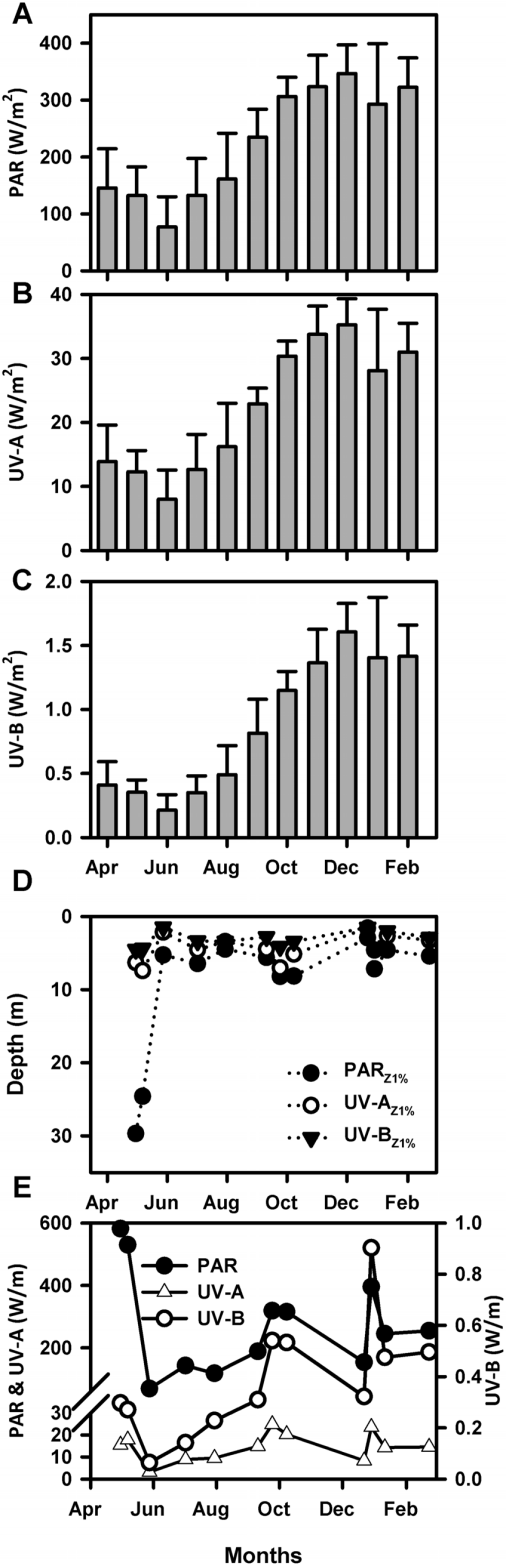
Solar Radiation Time Series. A) Time Series of average values of incident solar radiation for PAR (n = 7), B) UV-A (n = 7) and C) UV-B (n = 7) measured at noon in central Chile (April and February 2012). D) Depth penetration (Z_1%_) of solar radiation at noon between May 2011 and February 2012 in the coastal area off central Chile. E) Time series of the intensity of incident radiation integrated between the surface and the depth of 1% irradiance penetration of PAR and UV-A (left axis) and UV-B (right axis).

The mean value per month of incident atmospheric PAR (700–400 nm), UV-A (400–320 nm) and UV-B (320–280 nm) radiation measured in the Concepcion area (36°S) is shown in Fig. 2A–C. Data for all spectra followed the expected seasonal trend with lower values occurring during autumn and winter (April to August 2011) compared to spring and summer (September 2011 to February 2012). The lowest values for PAR, UV-A and UV-B radiation were found in June (77.18±53.08 Wm^−2^, 8.00±4.55 Wm^−2^ and 0.21±0.12 Wm^−2^, respectively) while the maximum values of the study period were found in December 2011 (346.78±50.19 Wm^−2^, 35.23±4.09 Wm^−2^ and 1.61±0.22 Wm^−2^ for PAR, UV-A and UV-B, respectively). The intensity of UV-A was always higher than UV-B (up to 37 times). Mean intensity values of UV-A radiation in winter 2011 were 12.28±4.11 Wm^−2^, while in summer 2012 it reached 31.43±3.59 Wm^−2^. UV-B radiation, mean winter value for 2011 reached 0.35±0.14 Wm^−2^, while in summer 2012 it increased to 1.47±0.11 Wm^−2^.

The penetration of solar radiation in the water column (Z1%) at Coliumo Bay is shown in Fig. 2D. As expected, the monthly mean value of Z1% during the entire study period was higher for PAR radiation than for UV-A and UV-B (9.4 m, 4.4 m and 3.2 m respectively). For PAR, the maximum penetration was found in winter 2011 (29.7 m in May). This can be explained by the influence of freshwater from river discharge or precipitation that determines salinity conditions in the first 20 m of the water column during winter time [34]. Minimum penetration values were observed in summer and reached 2.9 m in December 2011. UV-A radiation also penetrated at a maximum depth of 7.4 m in winter (May 2011) and only 1.6 m in summer (December 2011). This was also the case for UV-B radiation, for which maximum penetration was found in spring (November 2011) with 5.1 m and summer (3.1±1.2 m) while the minimum was found in May (1.5 m).

Integrated solar radiation in the water column for PAR, UV-A and UV-B is reported in Fig. 2E. Values varied significantly during the study period with PAR showing maximum values in winter (max. 581.49 Wm^−1^ in May 2011) while UV-A radiation peaked in late spring (December 2011, 23.58 Wm^−1^) with a penetration level of 7.1 m within the period of highest incident radiation (35.23±4.09 Wm^−2^). Finally, the integrated intensity of UV-B was lower than for PAR and UV-A, and reached a maximum value at the end of spring (December, 0.90 Wm^−1^) which coincides with a period of intense incident UV-B radiation (1.61±0.22 Wm^−2^).

### b) Photoammonification rates from diatom-derived and marine DOM

**Figure 3:**
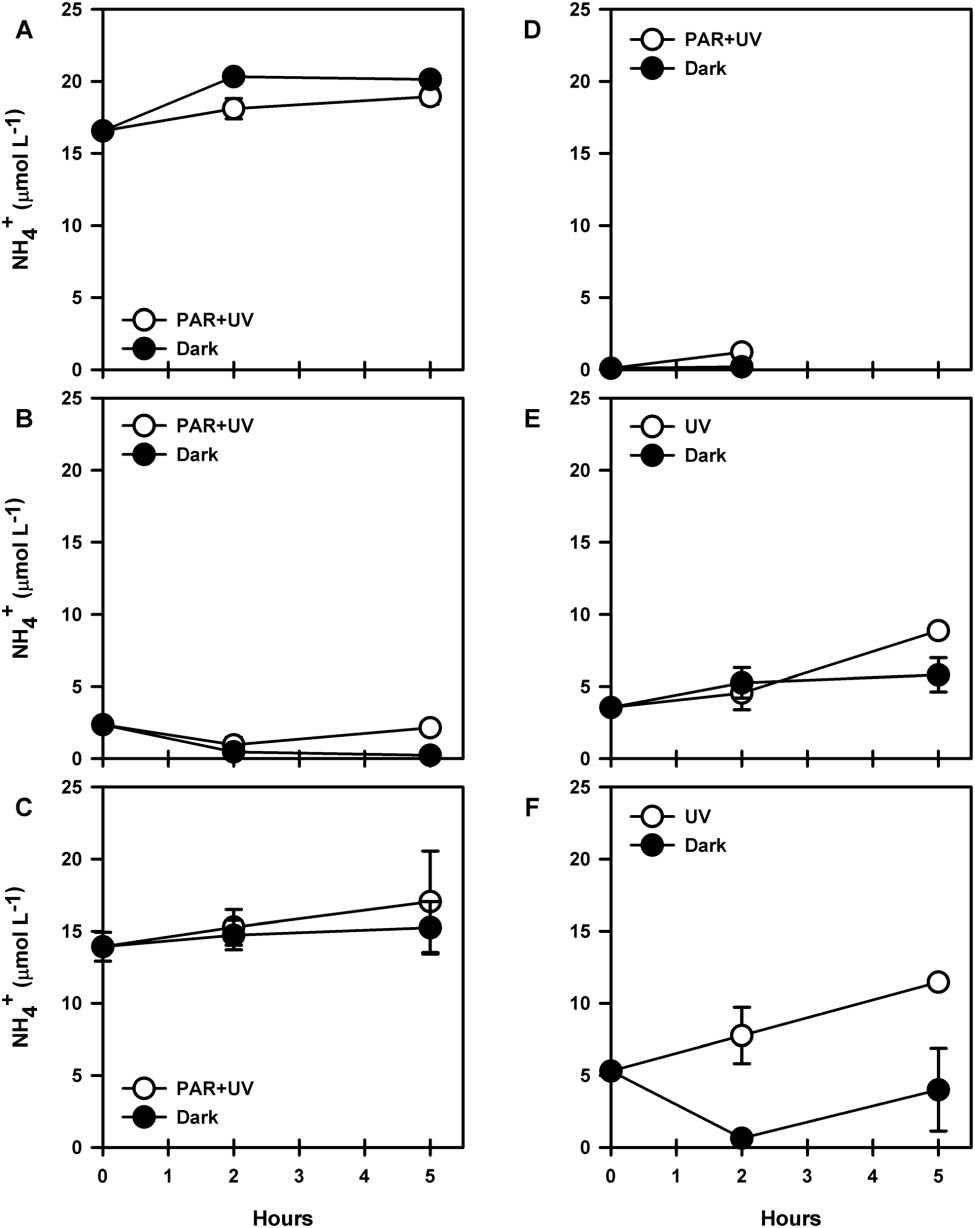
Evolution of ammonium concentrations during experiments of dissolved organic matter photodegradation using exudates of cultured *C. muelleri* at different cell densities (cell mL^−1^). A) 2.49E+06, B) 2.52E+06, C) 3.66E+06, D) 3.97E+06, E) 4.07E+06 and F) 4.44E+06. Error bar shows standard deviation for duplicate samples.

Figure 3 shows the ammonium evolution during photoammonification experiments in cultures of *C. muelleri*. Except for exudates derived from the lowest cell density, ammonium production was detected in all cases after exposure to PAR+UV or UV radiation only (Fig. 3A–F). Maximum ammonium concentrations were reached after 5 h of exposure and were always higher than dark control values (t-test, P = 0.001).

There was variability in ammonium concentrations at T0 due to differences in cell density at the moment of filtration. For instance, initial ammonium concentration for the experiments carried out in 23/04/2014 were 16.55±0.31 and 13.93±1.00 µmol L^−1^ for cell densities of 2.49E+06 and 3.66E+06 cell mL^−1^ respectively. The experiment carried out the day after 24/04/2014 showed an initial concentration of 3.56±0.21 µmol L^−1^ for cell density of 4.07E+06 cell mL^−1^. In spite of such variability, calculations of net ammonium photoproduction over time should not be affected by T0 concentrations.

For exudates from culture of diatom *C. muelleri* at density of 2.49E+06 cell mL^−1^ (April 2014; Fig. 1A) ammonium concentrations increased after exposure to PAR+UV radiation, both PAR+UV treatment and dark control. Although ammonium concentrations were higher in the dark control compared to PAR+UV treatment (t-test, P = 0.011), ammonium was produced in both cases at rates of 0.66 µmol L^−1 ^h^−1^ and 0.46 µmol L^−1 ^h^−1^ for dark and PAR+UV treatments, respectively.

Exposing exudates of *C. muelleri* from a higher cell density (February 2011; 2.52E+06 cell mL^−1^), to PAR+UV radiation (Fig. 3B) resulted in high ammonium concentrations compared to the dark control (t-test paired, P = 0.036). However, ammonium concentrations decreased during the incubation at a rate of 0.01 µmol L^−1 ^h^−1^ and 0.4 µmol L^−1 ^h^−1^ for PAR+UV-exposed and dark samples respectively (i.e. a 40 fold difference between both treatments). Ammonium photoproduction generated 0.12 µmol of NH_4_^+^ at a rate of 0.38 µmol L^−1 ^h^−1^ according to Eq. (2).

Exudates of *C. muelleri* obtained from cultures with higher cell density (April 2014; 3.66E+06 cell mL^−1^) showed high ammonium concentrations after exposure to PAR+UV radiation (Fig. 3C). Ammonium increased in the PAR+UV treatment at a rate of 0.62 µmol L^−1 ^h^−1^, higher than the rate obtained for the dark control (0.26 µmol L^−1 ^h^−1^). However, we did not find significant differences between PAR+UV treatment and dark control (paired t-test, P = 0.543). Ammonium photoproduction after 5 h of incubation could be estimated according to Eq. (2) as 0.36 µmol L^−1 ^h^−1^. In this case we estimated that 0.77 µmol of NH_4_^+^ were produced during the incubation.

High ammonium concentrations were also obtained using exudates of *C. muelleri* with higher cell densities (September 2011; 3.97E+06 cell mL^−1^). After exposure to PAR+UV radiation (Fig. 3D), ammonium concentrations were higher in samples exposed to PAR+UV compared to dark samples (paired t-test, P = 0.001). Ammonium was also produced in dark samples via microbial remineralization at a rate of 0.01 µmol L^−1 ^h^−1^. The estimated rate of ammonium photoproduction according to Eq. (2) reached 0.51 µmol L^−1 ^h^−1^. The amount of ammonium generated by photoproduction during the incubation reached 0.26 µmol.

In the case of exudates of *C. muelleri* at 4.07E+06 cell mL^−1^ (April 2014), exposure to UV radiation only produced high ammonium concentrations compared to the dark control (P = 0.010). Ammonium increased at rates of 1.09 and 0.43 µmol L^−1 ^h^−1^ for in both UV exposed samples and dark control, respectively. Ammonium photoproduction after 5 h of incubation was estimated according to Eq. (2) as 0.61 µmol L^−1 ^h^−1^.

At higher cell densities (4.44E+06 cell mL^−1^) ammonium production experiments (November 2011; Fig. 1F) resulted in higher concentrations in the treatment exposed to UVR than dark control (paired t-test, P = 0.004). Ammonium levels increased during the incubation in samples exposed to UVR at a rate of 1.23 µmol L^−1 ^h^−1^, while decreasing in the dark control at a rate of 0.15 µmol L^−1 ^h^−1^. The estimated rates of ammonium photoproduction of ammonium after 5 h of incubation according to Eq. (2) reached 1.49 µmol L^−1 ^h^−1^. The total amount of ammonium generated via photoammonification reached 2.24 µmol for this experiment.

**Figure 4:**
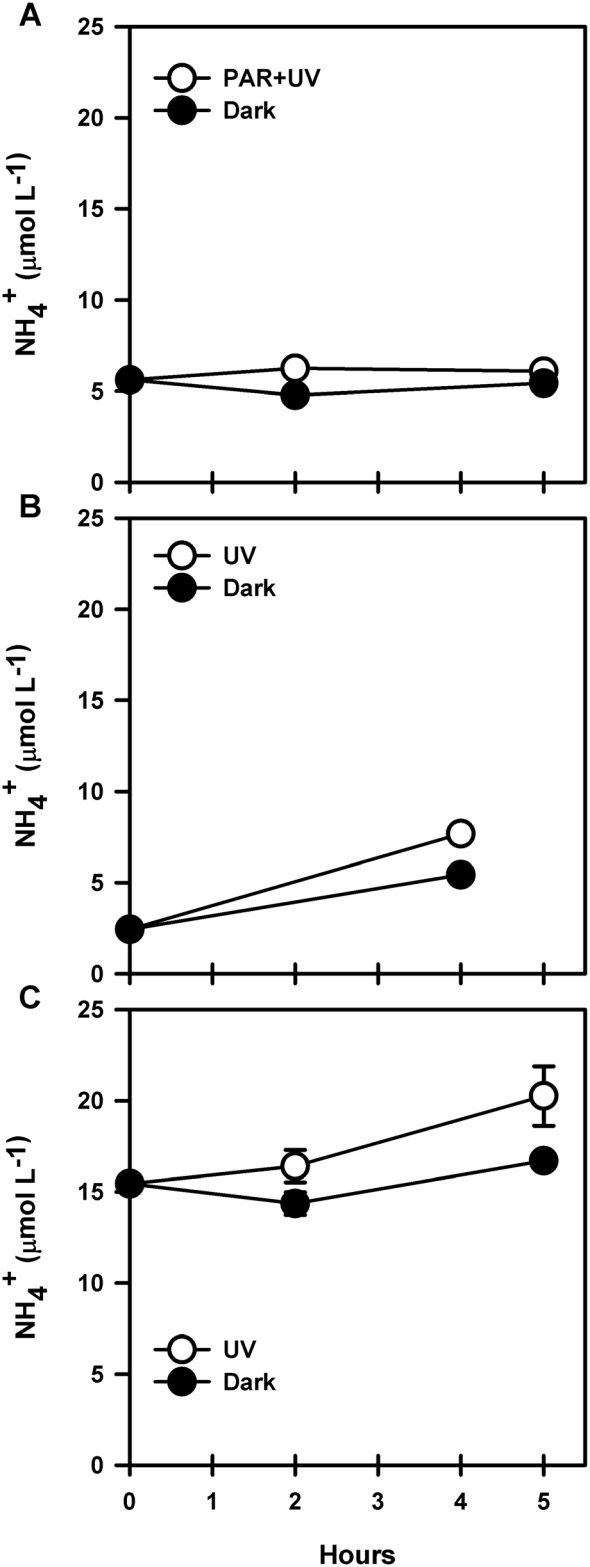
Evolution of ammonium concentrations during experiments of dissolved organic matter photodegradation using exudates of cultured *T. minuscule* at different cell densities. A) 2.82E+06, B) 3.41E+06, C) 3.65E+06 cell mL^−1^. Error bars show standard deviation for duplicate samples.

The variation of ammonium concentrations during exposure of exudates of *T. minuscule* coming from cultures with different cell densities to PAR+UV radiation is shown in Fig. 4. Higher ammonium concentrations were found in the exposed samples (DOM from cultures at 2.82E+06 cell mL^−1^) compared to dark conditions (paired t-test, P = 0.025). The observed ammonium production rate reached 0.08 µmol L^−1 ^h^−1^ in the treatment exposed to PAR+UV radiation, while in the dark control ammonium was consumed at a rate of 0.02 µmol L^−1 ^h^−1^. The estimated ammonium photoproduction rate after 5 h of incubation according to Eq. (2) was 0.13 µmol L^−1 ^h^−1^ and resulted in the generation of 0.19 µmol of NH_4_^+^.

Exposure of exudates of *T. minuscule* (3.41E+06 cell mL^−1^) to UVR led to higher ammonium concentrations compared to initial conditions (t-test, P<0.001, Fig. 4B). Ammonium production rates reached 1.31 µmol L^−1 ^h^−1^ and were lower in the dark control (0.75 µmol L^−1 ^h^−1^; Fig. 4B). Estimated ammonium photoproduction according to Eq. (2) reached 0.56 µmol L^−1 ^h^−1^ after 4 h of incubation. Ammonium generated by photoproduction was quantified as 0.67 µmol for this experiment.

Ammonium production was also observed in exudates of *T. minuscule* at higher cell densities (3.65E+06 cell mL^−1^) exposed only to UVR as shown in Fig. 4C. Ammonium concentrations were higher in the treatment exposed to UVR compared to dark conditions (paired t-test, P = 0.0242). The rate of ammonium production was 0.99 µmol L^−1 ^h^−1^ which is higher than the rate obtained for dark samples (0.29 µmol L^−1 ^h^−1^). Therefore, the estimated ammonium photoproduction (Eq. (2)) rate was 0.71 µmol L^−1 ^h^−1^ after 5 h of incubation. In this case production of ammonium generated 1.07 µmol during the experiment.

**Figure 5:**
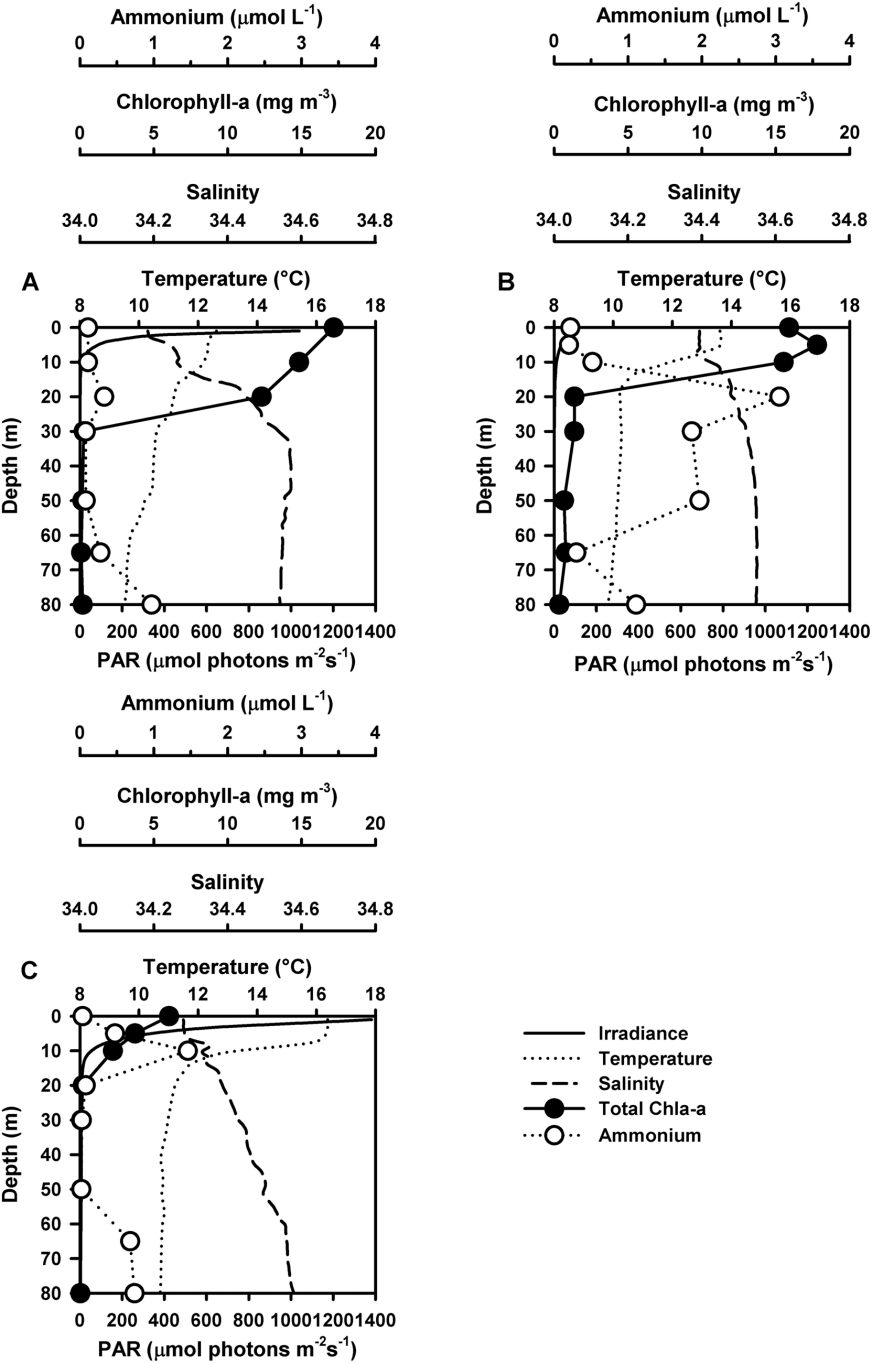
Hydrographic conditions of irradiance, salinity, temperature and ambient concentrations of total chlorophyll-a and ammonium during photoproduction experiments. Profiles were carried out in (A) September 2011, (B) November 2011 and (C) January 2012.

Three natural DOM photodegradation experiments were carried out in September, November 2011 and January 2012. Hydrographic conditions during sampling are shown in Fig. 5. During September 2011 (early spring), temperature varied between 12.6 in surface waters and 9.47°C in near bottom layers. The MLD was located at 15 m while Z1% was reached at 7 m depth. Total chlorophyll-concentrations reached 17.18 mg m^−3^ in near surface waters and rapidly decreased to values close to 0 at 30 m depth (Fig. 5A). Conditions during November 2011 (spring) showed higher SST values (13.65°C) and a shallow thermocline (10 m). Temperature in near bottom waters was close to 9.8°C (Fig. 5B). The mixed layer was 6 m deep while the euphotic zone (Z1%) reached 35 m depth. During January 2012 (summer), temperature varied from 16.4 to 10.7°C along the water column. A strong thermocline was located around 15 m. The depth of MLD was 7 m while the euphotic zone (Z1%) reached 15 m (Fig. 5C).

Ambient ammonium concentrations (Fig. 5) were higher in November 2011 compared to September 2011 and January 2012. During September 2011 (Fig. 5A) there were two subsurface maximum concentrations at 20 and 80 m (0.33 and 0.97 µmol L^−1^ respectively) while surface values reached 0.11 µmol L^−1^. In November 2011 (Fig. 5B), ambient ammonium in surface waters reached (0.22 µmol L^−1^). Subsurface concentrations were also high and reached 3.05 µmol L^−1^ at 20 m depth while near bottom water values were close to 1.1 µmol L^−1^. During January 2012 (Fig. 5C) surface ammonium concentration was close to 0.04 µmol L^−1^ and increased to 1.46 µmol L^−1^ at 10 m depth. Ammonium then decreased with depth reaching 0.02 µmol L^−1^ at 50 m. Values in near bottom waters showed a local increase that reached 0.74 µmol L^−1^.

**Figure 6:**
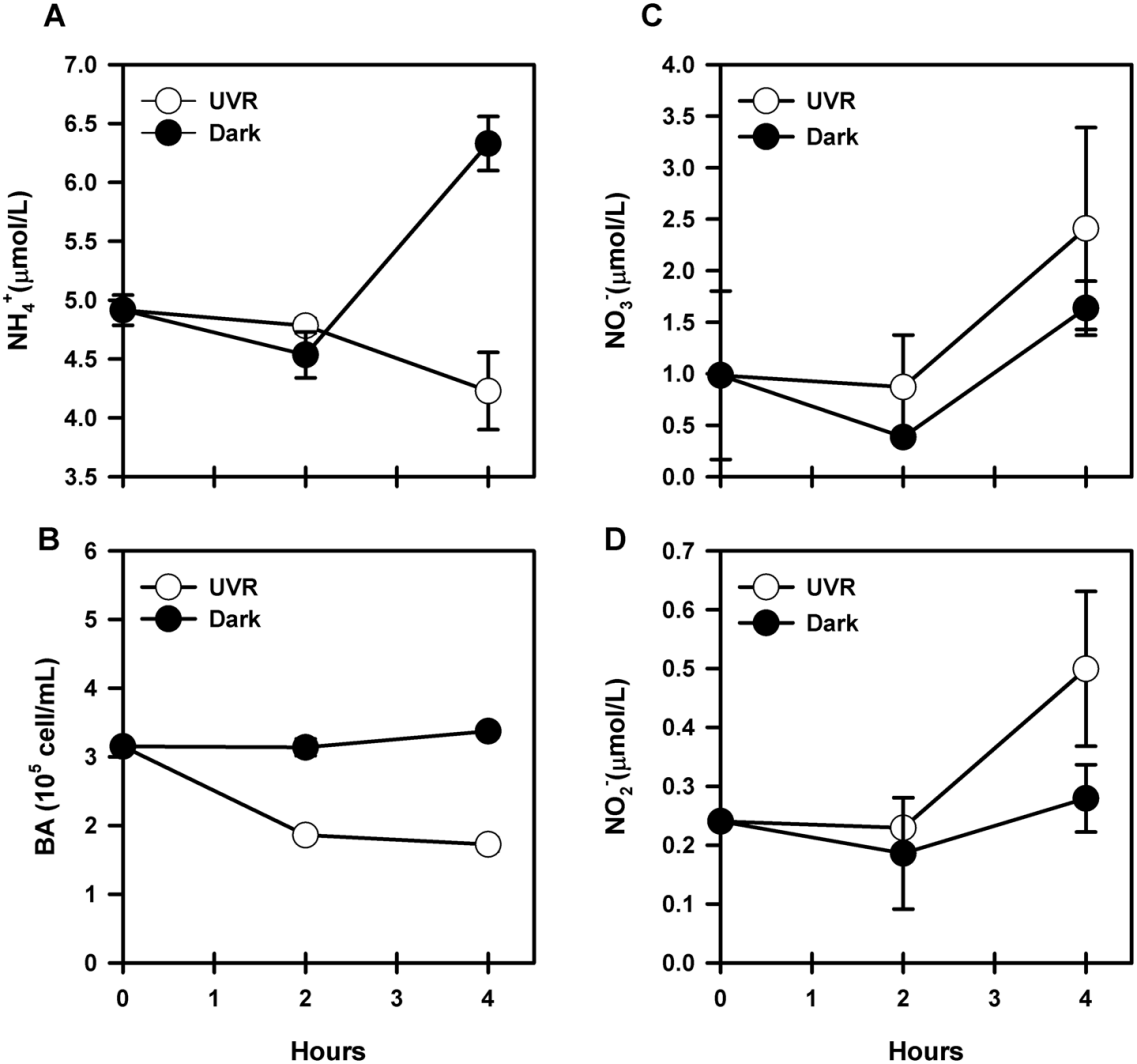
Evolution of nutrient concentration and bacterioplankton abundance during experiments of dissolved organic matter photodegradation (UVR vs Dark) using marine DOM (October 2011). A) Ammonium concentrations, B) bacterioplankton abundance (BA), C) Nitrate concentrations, D) Nitrite concentrations. All samples were taken in duplicate.

We evaluated ammonium production via photodegradation of marine DOM by exposure to UVR (UV-A+UV-B) during spring (November 2011; Fig. 6). Ammonium concentrations decreased during the first 2 h of exposure to UVR at a rate of 0.05 µmol L^−1 ^h^−1^ and reached concentrations of 4.78±0.06 µmol L^−1^ (Fig. 6A). After 4 h of exposure, concentrations continued to decrease and reached values close to 4.23±0.33 µmol L^−1^. Ammonium concentrations in dark samples decreased during the first 2 h of exposure but increased towards the end of the incubation period (4.54±0.19 µmol L^−1^ after 2 h and 6.33±0.23 µmol L^−1^ after 4 h). Interestingly, bacterioplankton abundance (Fig. 6B) was significantly lower in samples exposed to UVR compared to samples incubated in the dark (paired t-test, P = 0.002). Cell abundance after 2 h of exposure decreased in the samples exposed to UVR (1.86±0, 06×10^5^ cell mL^−1^), whereas dark samples showed no change compared to initial values (3.14±0.12×10^5^ cell mL^−1^). After 4 h of exposure, cell abundance increased in the dark control while decreasing in the sample exposed to UVR (3.37±0.08×10^5^ cell mL^−1^ and 1.72±0.10×10^5^ cell mL^−1^). Nitrate concentrations during these experiments increased in both UVR and dark treatments after 4 h of incubation. Concentrations varied between 1 µmol L^−1^ at T0 and 2.5 µmol L^−1^ for irradiated samples. Maximum concentrations for dark samples reached 1.7 µmol L^−1^ (Fig. 6C). Nitrite also showed an increase over time (Fig. 6D), but only for irradiated while remaining unchanged in dark samples. Values at T0 (0.25 µmol L^−1^) increased 2 fold after 4 h of incubation (0.5 µmol L^−1^). This suggests that ammonium consumption can be due to oxidizing activity by nitrifying microorganisms during our experiments.

**Figure 7:**
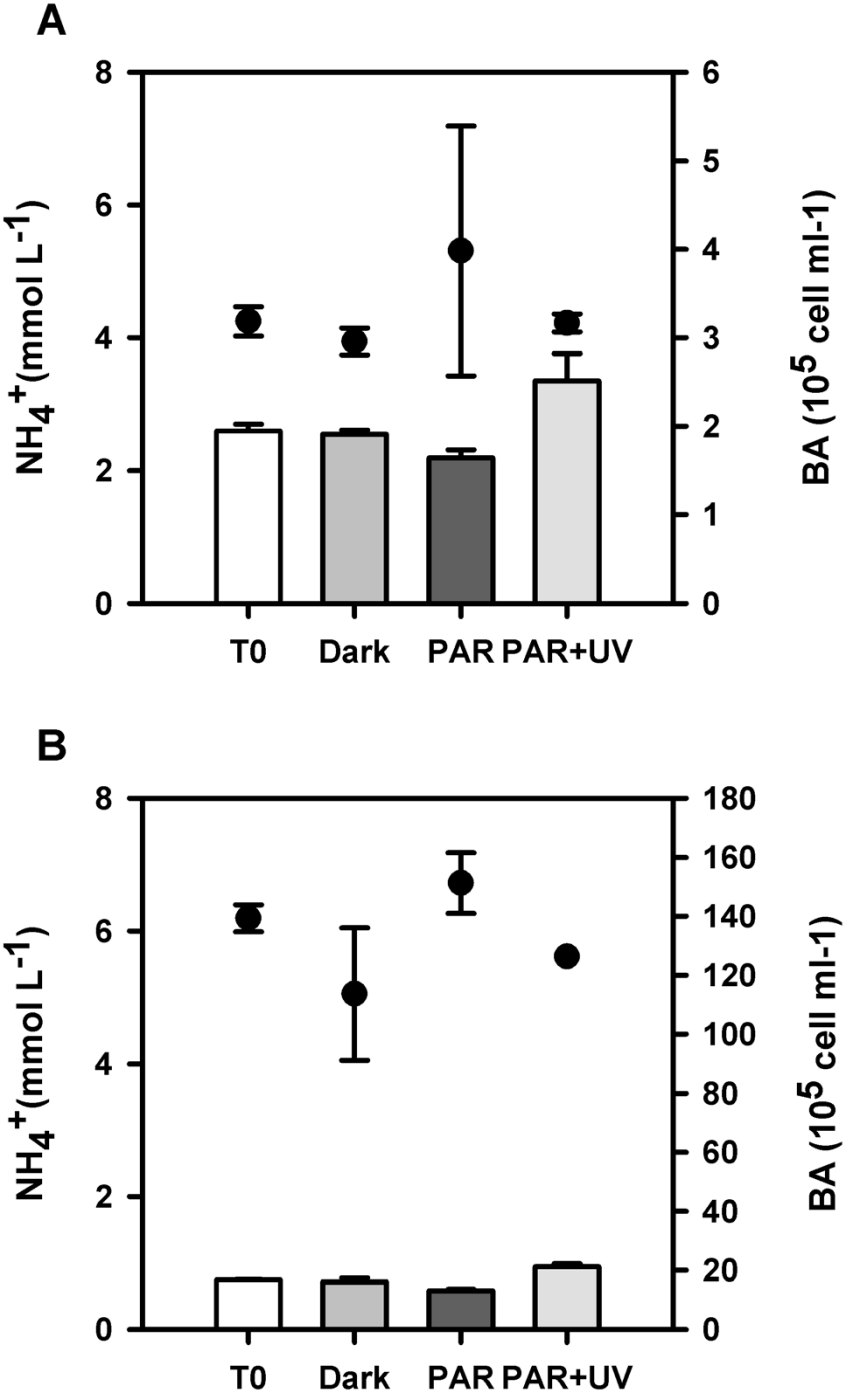
Evolution of ammonium concentrations during experiments of dissolved organic matter photodegradation (PAR+UVR vs PAR vs Dark) using marine DOM. Experiments carried out during A) September 2011 and B) January 2012. Vertical bars represent ammonium concentration per treatment. Bacterioplankton abundances (BA) are represented by circles. All samples were taken in duplicate.

Another set of experiments was carried out in spring (September 2011) and summer (January 2012; Fig. 7). Samples were exposed to total sunlight (PAR+UVR), PAR only or dark conditions. Results for September 2011 showed increasing ammonium concentrations increased in the samples exposed to PAR+UVR (3.35±0.41 µmol L^−1^) compared to samples exposed to PAR radiation only (2.19±0.12 µmol L^−1^; Tukey test, P<0.05). Following Eq. (2), we estimated the rate of ammonium photoproduction in the treatment exposed to PAR+UVR as 0.2 µmol L^−1 ^h^−1^. Bacterioplankton abundances in samples exposed to PAR radiation increased from 3.18±0.16×10^5^ cell mL^−1^ to 3.98±1.41×10^5^ cell mL^−1^ after 4 h of incubation. On the contrary, samples exposed to PAR+UVR as well as the dark control showed a decrease in cell counts over time (3.16±0.1×10^5^ cell mL^−1^ and 2.96±0.15×10^5^ cell mL^−1^, respectively), although no significant difference was found among all treatments (ANOVA one way, P = 0.7638).

The experiments carried out in summer (January 2012; Fig. 7B) also showed increasing ammonium concentrations in samples exposed to PAR+UV compared to initial conditions (0.94±0.04 µmol L^−1^ vs 0.75±0.003 µmol L^−1^) as well as PAR radiation only and dark conditions (Tukey test, P<0.05). Also, and as observed in our spring experiments described above, samples exposed to PAR radiation showed the lowest ammonium concentrations during the incubation (0.58±0.03 µmol L^−1^). The rate of photoammonification (Eq. (2)) for samples exposed to PAR+UVR reached 0.06 µmol L^−1 ^h^−1^. Bacterioplankton abundances during the incubation (Fig. 7B) only showed an increase in the treatment exposed to PAR radiation (151±10×10^5^ cell mL^−1^ vs 139±5×10^5^ cell mL^−1^) while values decreased in samples exposed to PAR+UVR as well as the dark control (126±0 and 114±22×10^5^ cell mL^−1^, respectively). In spite of the observed differences, results between treatments were not significantly different between each other (one way ANOVA, P = 0.158).

## Discussion

Our incident radiation data represents a seasonal survey of the study area that complements previous measurements [30]. The results show that irradiance levels increased 2.6, 2.6 and 4.2 times for PAR, UV-A and UV-B respectively in summer compared to winter, following a coherent seasonal trend.

The depth of penetration of solar radiation (as PAR, UV-A and UV-B) between May 2011 and February 2012 varied in the study area, with a decrease in values between autumn 2011 and summer 2012. Maximum penetration in the water column was observed in autumn (April and May 2011), which coincides with the end of active upwelling conditions in the study area [35]. During this time, turbidity decreases in the water column allowing deeper penetration of solar radiation. Also important is the influence of freshwater inputs (as precipitation and river discharge) in the first meters of the water column [34] which modifies its optical properties compared to spring and summer. Nevertheless, UVR (both UV-A and UV-B) was measurable in the coastal area off central Chile during the entire study period (Fig. 2), suggesting a potential year-round occurrence of photochemical transformation of DOM in the first meters of the water column. Values of depth penetration (Z1%) reached 29.7 m for PAR, 7.4 m for UV-A and 5.1 m for UV-B. Although PAR Z1% seems deeper than expected for a coastal upwelling system [29], it is coherent with time data gathered historically at station 18 (unpublished data COPAS Time Series program).

Although a few available data sets exist for the study area and are complementary with our results, they may not be entirely comparable because of the well-known differences between the instruments used. Our average UV-A values for winter 2011 are lower than data previously reported for 2003–2004 [30]. Our average UV-B values for winter and summer were also 3–4 times lower than a previous study [30]. A latitudinal comparison shows that UV-A (320–400 nm) values reported for lower and higher latitudes in Chile (Santiago, 33°33′ and Punta Arenas, 53°08′) are close to our values but we reports lower values of UV-B (280–320 nm) for the same latitudes [36]. The estimated penetration of solar radiation during this study is also lower than previous measurements in open ocean waters at the same latitude (36°S; 74°W) [7] while they are in the same range of estimations as Z10% reported for Seno Reloncaví and Valdivia in southern Chile [37]. However, since our Z1% estimations include the assumption of a homogeneous water column [3] and values of integrated solar radiation are affected by the intensity of incident solar radiation and the optical properties of the water, comparing our results with existing data must be done carefully. As stated previously, the variety of instruments used for underwater measurement of UVR precludes further comparisons [29].

High integrated values obtained in this study (up to 581, 25 and 1 W m^−1^ of PAR, UV-A and UV-B respectively, Fig. 2E) corresponded to the deepest values of Z1% (in autumn) while in summer, when the mixed layer heat balance is dominated by solar radiation [34], high integrated values responded to higher intensity of incident radiation but not deeper Z1%. This can be explained by the increased turbidity of the water column during the productive season which combines biological particles (phytoplankton and bacterioplankton) and high sedimentation rates previously observed in the study area [38].

We also report a persistent exposure during the year of the first meters of the water column to UV-A and UV-B. The effects of incident solar radiation have consequences for the photoproduction of bioavailable compounds for bacterioplankton [9] and can also have negative effects such as DNA damage [4], inhibition of photosynthesis [39], [40] and decrease of bacterioplankton production [5]. However, the persistent exposure of the euphotic layer to UVR has also implications for biogeochemical processes which may result in increased availability of ammonium (derived from photodegradation of DOM) as well as enhanced heterotrophic N utilization and primary production. Further direct measurements of the optical properties of the water column are therefore needed to complement these results, including the characterization of chromophoric dissolved organic matter (CDOM) and its distribution, which is a major contributor to the attenuation of solar radiation in the ocean [3].

We evaluated the photoproduction of ammonium using DOM obtained from diatom cultures and natural samples (Time series coastal st 18). In this process, the exposure to sunlight (primarily UV radiation, [41]) causes the breakdown DOM and the consequent release of NH_4_^+^ [15], [19], [42], [43]. Photoproduction of ammonium was observed in all cases (Table 3), in agreement with previous studies (Table 4).

**Table 3:** Summary of ammonium production rates (expressed in µmol L^−1 ^h^−1^) obtained for diatom-derived DOM and marine DOM (seawater filtered through 0.7 µm) during this study.

Type of sample	Date	Range of exposure	Exposure time (h)	Net NH_4_^+^ photoproduction (µmol L^−1 ^h^−1^)
*C. muelleri*	23/04/2014	PAR+UVR	5	N.D
*C. muelleri*	11/10/2011	PAR+UVR	5	0.38
*C. muelleri*	23/04/2014	PAR+UVR	5	0.36
*C. muelleri*	21/09/2011	PAR+UVR	2	0.51
*C. muelleri*	24/04/2014	UVR	5	0.61
*C. muelleri*	11/11/2011	UVR	5	1.49
*T. minuscule*	11/10/2011	PAR+UVR	5	0.13
*T. minuscule*	23/01/2012	UVR	4	0.56
*T. minuscule*	11/11/2011	UVR	5	0.71
Marine DOM	13/09/2011	PAR+UVR	4	0.2
Marine DOM	11/1/2012	PAR+UVR	4	0.06
Marine DOM	21/11/2011	UVR	4	N.d

PAR: Photosynthetically active radiation. UVR: UV-A+UV-B. N.D: Not Detected.

**Table 4:** Comparison between rates of photoammonification obtained from the literature for freshwater, estuarine and marine environments and results obtained from this study (µmol L^−1 ^h^−1^).

Type of sample	Photoammonification rate (µmol L^−1 ^h^−1^)	References
Freshwater, estuarine isolated humics	0.05–0.37	Bushaw et al., 1996 [12]
Freshwater, estuarine isolated humics	0.1	Gao & Zeep, 1998 [15]
Freshwater	0	Bertilson et al., 1999 [21]
Coastal lagoon	−0.29	Gardner et al., 1998 [42]
Estuarine	0.007–0.06	Bushaw-Newton & Moran, 1999 [43]
Coastal lagoon	0.001–0.046	Buffam & McGlathery, 2003 [19]
Marine, filtered by 0.1 µm	0.0004–0.0029	Kitidis et al., 2006 [14]
Exudates *C. muelleri* filtered by 0.7 µm	0.36–1.49	this study
Exudates *T. minuscule* filtered by 0.7 µm	0.13–0.71	this study
Marine, filtered by 0.7 µm	0.06–0.20	this study

Diatom-derived DOM exposed to PAR+UVR and UVR only resulted in ammonium production in two different types of cultures: *C. muelleri* and *T. minuscule* (Table 3). Because cultures were filtered through 0.7 µm, we assume that some bacterial activity (as ammonium consumption) may occur. This is particularly relevant for explaining the variability in T0 ammonium concentrations observed although the main factor influencing T0 variability may come from the differences in cell densities used in the cultures (Table 1). Nevertheless, ammonium was produced in samples exposed to PAR+UVR as well as in samples exposed to UVR only (the latter showing higher rates than values for PAR+UVR exposure). Rates of photoproduction of ammonium for *C. muelleri* was estimated at 0.38, 0.36 and 0.51 µmol L^−1 ^h^−1^ while exposed to PAR+UVR. Samples exposed to UVR only generated higher ammonium levels (0.61 and 1.49 µmol L^−1 ^h^−1^). Exudates of *T. minuscule* exposed to PAR+UVR resulted in the generation of ammonium at a rate of 0.13 µmol L^−1 ^h^−1^. Rates obtained for samples exposed to UVR only were higher and reached 0.56 and 0.71 µmol L^−1 ^h^−1^. The rates of ammonium production obtained from DOM exudates from diatom cultures showed variability associated not only to the origin of DOM but also to its quality, a variable approached in this case by the use of cultures at different cell densities. In general, higher photoammonification rates were obtained with higher cell densities, which support the occurrence of photoammonification in high productivity regions such as the study area.

Photoproduction of ammonium was indeed observed during exposure of marine DOM to solar radiation (Table 3). According to culture-DOM results, ammonium photoproduction mainly occurred during the most productive seasons, spring (0.2 µmol L^−1 ^h^−1^) and summer (0.06 µmol L^−1 ^h^−1^) and mainly under PAR+UVR exposure. Interestingly, no net production of ammonium was observed during exposure to UVR only or PAR only. In fact, exposure to PAR resulted in increased ammonium consumption compared to dark and initial conditions. Accordingly, bacterioplankton abundance increased in samples exposed to PAR only compared to PAR+UVR, therefore enhancing ammonium consumption. On the other hand, exposure to UVR only resulted in ammonium consumption and decreasing bacterial abundance while increasing nitrite and nitrate concentrations were measured. This suggests active ammonium and nitrite oxidation in spite of UV exposure. This fact could explain the lower rates of ammonium photoproduction obtained for natural marine samples compared to cultured DOM. Consequently, the absence of ammonium production in samples exposed to PAR and the occurrence of ammonium production under PAR+UVR exposure suggest that active photoammonification can take place under full sunlight while PAR exposure enhances its biological consumption. This has been observed for microplankton, which can use UV-A as a source of energy as well as PAR for carbon fixation [44] while UV-B has an inhibitory effect. Furthermore, light stress (defined as high exposure to UV-B) seems to have enhanced inhibitory effects under nutrient limitation (particularly nitrate [45]), which is seldom observed in surface waters of our study area.

Our rates of ammonium photoproduction using marine DOM are smaller [12], [15] or in the range [14], [19], [43] of results previously reported for marine waters (Table 4). They are nevertheless higher than rates obtained for oligotrophic and coastal lagoon waters [14], [19]. This is coherent with previous observations of lower efficiency in photoammonification for coastal compared to off shore waters. Efficiency of photoproduction of ammonium is also dependent of the molar C:N ratio of DOM, which can explain the higher rates obtained with diatom exudates compared to naturally occurring DOM [46]. Also, at high ratios of bacterial activity and DOC, exposure to UVR does not necessarily result in increased availability of organic matter [8].

It is known that photochemical transformation of DOM can support heterotrophic and autotrophic plankton [41] and also increase bacterial production and abundance [9]. In order to determine the potential contribution of ammonium photoproduction in our experiments to local primary production we used phytoplankton uptake data reported for st.18 [26]. We estimated that the daily ammonium photoproduction from *C. muelleri* and *T. minuscule* exudates could support between 177 and 456% of the phytoplankton NH_4_^+^ demand in spring and summer, respectively. Photoproduction of ammonium from natural DOM on the other hand has the potential for supporting between 50 and 178% of spring-summer phytoplankton demand [26]. Importantly, they could also sustain 100% of chemoautotrophic bacterial ammonium oxidation (the first step of the nitrification process) which can reach rates close to 50 nmol L^−1 ^d^−1^ in surface waters of the study area [26].

These values are higher than the potential contribution of photochemically produced N to phytoplankton new production in the Baltic Sea where N deprived communities directly react to bioavailable N [41]. However, photoammonification and biological ammonium regeneration cannot be dissociated, the latter acting under full sunlight exposure and co-occurring with the former. Photoammonification has been suggested to be strong in shelf waters and marginal seas [14]. It has also been shown to decrease net community metabolism off the Eastern South Pacific [7]. Our results demonstrate that ammonium photoproduction under PAR+UVR occurs off central Chile in combination with its biological regeneration and consumption. Because net ammonium photoproduction is not usually accounted for in regional nitrogen budgets, our results have important implications for the understanding of mechanisms sustaining primary production in this coastal upwelling system.

## Conclusions

We report a year-round influence of UVR in the first 2 to 7 meters of the water column in the coastal zone off central Chile. Net ammonium photoproduction via degradation of representative diatom exudates and natural DOM samples represents a potential contribution to the local nitrogen budget at short times scales (2 or 5 h). Photoammonification in culture-derived DOM was variable but showed higher rates in samples derived from higher cell density cultures. Naturally occurring DOM exposed to PAR+UVR resulted in net photoammonification at rates ranging between 0.06 and 0.2 µmol L^−1 ^h^−1^. No photoproduction of ammonium was observed after exposure to PAR, but increased consumption and bacterial abundance was observed.

Based in existing phytoplankton N uptake data for the study area [26], we estimate that net ammonium photoproduction could support between 50 and 178% of spring-summer phytoplankton NH_4_^+^ demand.

## References

[1] Manney GL, Santee ML, Rex M, Livesey NJ, Pitts MC, et al (2011) Unprecedented Arctic ozone loss in 2011. Nature 478: 469–475. 21964337 10.1038/nature10556

[2] WMO (2011) Scientific Assessment of Ozone Depletion: 2010, Global Ozone Research and Monitoring Project–Report No. 52. Geneva, Switzerland. 516 p.

[3] Whitehead R, de Mora S, Demers S (2000) Enhanced UV radiation–a new problem for the marine environment. In: de Mora S, Demers S, Vernet M, editors. The effects of UV radiation in the marine environment. Cambridge. 1–34.

[4] Helbling WE, Buma AGJ, Boer MKd, Villafañe VE (2001) In situ impact of solar ultraviolet radiation on photosynthesis and DNA in temperate marine phytoplankton. Mar Ecol Prog Ser 211: 43–49.

[5] Hernández KL, Quiñones RA, Daneri G, Helbling EW (2006) Effects of solar radiation on bacterioplankton production in the upwelling system off central-southern Chile. Mar Ecol Prog Ser 315: 19–31.

[6] Hader DP, Kumar HD, Smith RC, Worrest RC (2007) Effects of solar UV radiation on aquatic ecosystems and interactions with climate change. Photochem Photobiol Sci 6: 267–285. 17344962 10.1039/b700020k

[7] Godoy N, Canepa A, Lasternas S, Mayol E, Ruíz-Halpern S, et al (2012) Experimental assessment of the effect of UVB radiation on plankton community metabolism along the Southeastern Pacific off Chile. Biogeosciences 9: 1267–1276.

[8] Obernosterer I, Reitner B, Herndl GJ (1999) Contrasting effects of solar radiation on dissolved organic matter and its bioavailability to marine bacterioplankton. Limnol Oceanogr 44: 1645–1654.

[9] De Lange HJ, Morris DP, Williamson CE (2003) Solar ultraviolet photodegradation of DOC may stimulate freshwater food webs. J Plankton Res 25: 111–117.

[10] Abboudi M, Jeffrey WH, Ghiglione JF, Pujo-Pay M, Oriol L, et al (2008) Effects of photochemical transformations of dissolved organic matter on bacterial metabolism and diversity in three contrasting coastal sites in the Northwestern Mediterranean Sea during summer. Microb Ecol 55: 344–357. 17674086 10.1007/s00248-007-9280-8

[11] Pakulski JD, Baldwin A, Dean AL, Durkin S, Karentz D, et al (2007) Responses of heterotrophic bacteria to solar irradiance in the eastern Pacific Ocean. Aquat Microb Ecol 47: 153.

[12] Bushaw KL, Zepp RG, Tarr MA, Schulz-Jander D, Bourbonniere RA, et al (1996) Photochemical release of biologically available nitrogen from aquatic dissolved organic matter. Nature 381: 404–407.

[13] Mopper K, Kieber DJ (2002) Photochemistry and the cycling of carbon, sulfur, nitrogen and phosphorus. In: Hansell D, A, Carlson C, A, editors. Biogeochemistry of marine dissolved organic matter. 455–507.

[14] Kitidis V, Uher G, Upstill-Goddard R, Mantoura R, Spyres G, et al (2006) Photochemical production of ammonium in the oligotrophic Cyprus Gyre (Eastern Mediterranean). Biogeosciences 3: 439–449.

[15] Gao H, Zepp RG (1998) Factors influencing photoreactions of dissolved organic matter in a coastal river of the southeastern United States. Environ Sci Technol 32: 2940–2946.

[16] Berman T, Béchemin C, Maestrini SY (1999) Release of ammonium and urea from dissolved organic nitrogen in aquatic ecosystems. Aquat Microb Ecol 16: 295–302.

[17] Kieber RJ, Li A, Seaton PJ (1999) Production of nitrite from the photodegradation of dissolved organic matter in natural waters. Environ Sci Technol 33: 993–998.

[18] Wang W, Tarr MA, Bianchi TS, Engelhaupt E (2000) Ammonium photoproduction from aquatic humic and colloidal matter. Aquat Geochem 6: 275–292.

[19] Buffam I, McGlathery KJ (2003) Effect of ultraviolet light on dissolved nitrogen transformations in coastal lagoon water. Limnol Oceanogr 48: 723–734.

[20] Koopmans DJ, Bronk DA (2002) Photochemical production of dissolved inorganic nitrogen and primary amines from dissolved organic nitrogen in waters of two estuaries and adjacent surficial groundwaters. Aquat Microb Ecol 26: 295–304.

[21] Bertilsson S, Stepanauskas R, Cuadros-Hansson R, Graneli W, Wikner J, et al (1999) Photochemically induced changes in bioavailable carbon and nitrogen pools in a boreal watershed. Aquat Microb Ecol 19: 47–56.

[22] Morell JM, Corredor JE (2001) Photomineralization of fluorescent dissolved organic matter in the Orinoco River plume: Estimation of ammonium release. Journal of Geophysical Research-Oceans 106: 16807–16813.

[23] Daneri G, Dellarossa V, Quiñones R, Jacob B, Montero P, et al (2000) Primary production and community respiration in the Humboldt Current System off Chile and associated oceanic areas. Mar Ecol Prog Ser 197: 41–49.

[24] Dugdale RC, Goering JJ (1967) Uptake of new and regenerated forms of nitrogen in primary productivity. Limnol Oceanogr 12: 196–206.

[25] Fernández C, Farías L, Alcaman M (2009) Primary production and nitrogen regeneration processes in surface waters of the Peruvian upwelling system. Prog Oceanogr 83: 159–168.

[26] Fernandez C, Farías L (2012) Assimilation and regeneration of inorganic nitrogen in a coastal upwelling system: ammonium and nitrate utilization. Mar Ecol Prog Ser 451: 1–14.

[27] Farías L, Fernández C, Faúndez J, Cornejo M, Alcaman ME (2009) Chemolithoautotrophic production mediating the cycling of the greenhouse gases N2O and CH4 in an upwelling ecosystem. Biogeosciences 6: 3053–3069.

[28] Escribano R, Schneider W (2007) The structure and functioning of the coastal upwelling system off central/southern Chile. Progress In Oceanography 75: 343–347.

[29] Tedetti M, Sempéré R (2006) Penetration of ultraviolet radiation in the marine environment. A review. Photochem Photobiol 82: 389–397. 16613490 10.1562/2005-11-09-IR-733

[30] Hernández K, Yannicelli B, Montecinos A, Ramos M, González HE, et al (2012) Temporal variability of incidental solar radiation and modulating factors in a coastal upwelling area (36°S). Prog Oceanogr 92: 18–32.

[31] Holmes RM, Aminot A, Kérouel R, Hooker BA, Peterson BJ (1999) A simple and precise method for measuring ammonium in marine and freshwater ecosystems. Can J Fish Aquat Sci 56: 1801–1808.

[32] de Boyer Montegut C, Madec G, Fischer AS, Lazar A, Iudicone D (2004) Mixed layer depth over the global ocean: An examination of profile data and a profile-based climatology. Journal of Geophysical Research: Oceans (1978–2012) 109.

[33] Marie D, Simon N, Guillou L, Partensky F, Vaulot D (2000) Flow cytometry analysis of marine picoplankton. In Living Color: Springer. 421–454.

[34] Sobarzo M, Bravo L, Donoso D, Garces-Vargas J, Schneider W (2007) Coastal upwelling and seasonal cycles that influence the water column over the continental shelf off central Chile. Prog Oceanogr 75: 363–382.

[35] Sobarzo M, Djurfeldt L (2004) Coastal upwelling process on a continental shelf limited by submarine canyons, Concepción, central Chile. Journal of Geophysical Research: Oceans (1978–2012) 109.

[36] Cabrera S, Bozzo S, Fuenzalida H (1995) Variations in UV radiation in Chile. J Photochem Photobiol B 28: 137–142. 7636634 10.1016/1011-1344(94)07103-u

[37] Huovinen P, Gómez I (2011) Spectral attenuation of solar radiation in Patagonian fjord and coastal waters and implications for algal photobiology. Cont Shelf Res 31: 254–259.

[38] Montero P, Daneri G, Cuevas LA, González HE, Jacob B, et al (2007) Productivity cycles in the coastal upwelling area off Concepción: The importance of diatoms and bacterioplankton in the organic carbon flux. Prog Oceanogr 75: 518–530.

[39] Arrigo KR, Lubin D, van Dijken GL, Holm-Hansen O, Morrow E (2003) Impact of a deep ozone hole on Southern Ocean primary production. J Geophys Res 108: 3154.

[40] Holm-Hansen O, Villafañe VE, Helbling EW (1997) Effects of solar ultraviolet radiation on primary production in Antarctic waters. In: Battaglia B, Valencia J, Walton DWH, editors. Antarctic communities: Species, structure and survival. Cambridge: Cambridge University Press. 375–380.

[41] Vahatalo A, Jarvinen M (2007) Photochemically produced bioavailable nitrogen from biologically recalcitrant dissolved organic matter stimulates production of a nitrogen-limited microbial food web in the Baltic Sea. Limnol Oceanogr 52: 132.

[42] Gardner WS, Cavaletto JF, Bootsma HA, Lavrentyev PJ, Troncone F (1998) Nitrogen cycling rates and light effects in tropical Lake Maracaibo, Venezuela. Limnol Oceanogr 43: 1814–1825.

[43] Bushaw-Newton KL, Moran MA (1999) Photochemical formation of biologically available nitrogen from dissolved humic substances in coastal marine systems. Aquat Microb Ecol 18: 285–292.

[44] Gao K, Li G, Helbling EW, Villafane VE (2007) Variability of UVR effects on photosynthesis of summer phytoplankton assemblages from a Tropical Coastal Area of the South China Sea†. Photochem Photobiol 83: 802–809. 17645650 10.1111/j.1751-1097.2007.00154.x

[45] Bouchard J, Longhi M, Roy S, Campbell D, Ferreyra G (2008) Interaction of nitrogen status and UVB sensitivity in a temperate phytoplankton assemblage. Journal of Experimental Marine Biology and Ecology 359: 67–76.

[46] Xie H, Bélanger S, Song G, Benner R, Taalba A, et al (2012) Photoproduction of ammonium in the southeastern Beaufort Sea and its biogeochemical implications. Biogeosciences Discussions 9: 4441–4482.

